# Assistive Technology Use and Provision During COVID-19: Results From a Rapid Global Survey

**DOI:** 10.34172/ijhpm.2020.210

**Published:** 2020-11-11

**Authors:** Emma M. Smith, Maria Luisa Toro Hernandez, Ikenna D. Ebuenyi, Elena V. Syurina, Giulia Barbareschi, Krista L. Best, Jamie Danemayer, Ben Oldfrey, Nuha Ibrahim, Catherine Holloway, Malcolm MacLachlan

**Affiliations:** ^1^Assisting Living and Learning Institute, Maynooth University, Maynooth, Ireland.; ^2^Independent Researcher, Madrid, Spain.; ^3^Athena Institute, Vrije Universiteit, Amsterdam, The Netherlands.; ^4^Global Disability Innovation Hub, University College London, London, UK; ^5^Center for Interdisciplinary Research in Rehabilitation and Social Integration, Universite Laval, Québec, QC, Canada.; ^6^Centre for Global Health, Trinity College Dublin, Dublin 2, Ireland.; ^7^University College London Interaction Centre, University College London, London, UK.; ^8^Assisting Living and Learning Institute, Department of Psychology, Maynooth University, Maynooth, Ireland.

**Keywords:** Assistive Technology, Health Policy, Ageing, Disability, Resilience, Crises

## Abstract

**Background:** The coronavirus disease 2019 (COVID-19) pandemic has impacted all segments of society, but it has posed particular challenges for the inclusion of persons with disabilities, those with chronic illness and older people regarding their participation in daily life. These groups often benefit from assistive technology (AT) and so it is important to understand how use of AT may be affected by or may help to mitigate the impacts of COVID-19.

**Objective: **The objectives of this study were to explore the how AT use and provision have been affected during the initial stages of the COVID-19 pandemic, and how AT policies and systems may be made more resilient based on lessons learned during this global crisis.

**Methods:** This study was a rapid, international online qualitative survey in the 6 United Nations (UN) languages (English, French, Spanish, Russian, Arabic, Mandarin Chinese) facilitated by extant World Health Organization (WHO) and International Disability Alliance networks. Themes and subthemes of the qualitative responses were identified using Braun and Clarke’s 6-phase analysis.

**Results: **Four primary themes were identified in in the data: Disruption of Services, Insufficient Emergency Preparedness, Limitations in Existing Technology, and Inadequate Policies and Systems. Subthemes were identified within each theme, including subthemes related to developing resilience in AT systems, based on learning from the pandemic.

**Conclusion: **COVID-19 has disrupted the delivery of AT services, primarily due to infection control measures resulting in lack of provider availability and diminished one-to-one services. This study identified a need for stronger user-centred development of funding policies and infrastructures that are more sustainable and resilient, best practices for remote service delivery, robust and accessible tools and systems, and increased capacity of clients, caregivers, and clinicians to respond to pandemic and other crisis situations.

## Background

**Key Messages**

** Implications for policy makers**

- Service users must be engaged in development of sustainable policies and infrastructures which meet their needs using a co-created rights-based approach.
- Service providers must develop their skills and capacity for delivery of remote services and provide training and appropriate digital and assistive technologies to clients and their caregivers to facilitate engagement with health services and their communities.
- Product manufacturers must work with service users to understand their needs for digital and assistive technologies, and develop robust and accessible tools for service delivery.
- Assistive technology (AT) must be recognized as an essential service, and must be accompanied by best practices and funding models for remote service delivery.
- Policies and infrastructures must be developed which recognize access to AT as key to the realization of rights for persons who require them and must include AT within a universal health coverage system.

** Implications for the public**

Assistive technologies may be used by any person with a limitation in their daily activities – from temporary disability due to injury or illness, chronic conditions, physical or psychosocial disability, or ageing. In crisis situations, such as the coronavirus disease 2019 (COVID-19) pandemic, access to these devices and associated services, including training, maintenance, and repair may be impacted. This research supports access to assistive technologies through the development of sustainable policies and processes which ensure all who require assistive technologies to maintain independence are able to access the necessary products and services.

The coronavirus disease 2019 (COVID-19) pandemic has impacted individuals’ participation in daily activities including community mobility, access to education and employment, and access to healthcare.^1^ While these experiences have been felt globally the COVID-19 pandemic has introduced additional vulnerability and marginalization to those with some type of functional impairment – people with disabilities, chronic illness or frailty due to ageing.^2-6^ Evidence suggests, even in non-pandemic times, persons with disabilities experience lower socioeconomic status, lower rates of employment, lower overall health status, and higher rates of poverty.^7-12^ In the context of a pandemic, many of these factors contribute to the classification of these individuals as vulnerable people, which may further result in marginalization from society in the name of protection from illness.^2^ This cycle of oppression, marginalization, and exclusion has direct impact on the realization of rights for persons with disabilities as enshrined in the United Nations Convention on the Rights of Persons with Disabilities (UNCRPD).^13^

 To enable full access to rights and opportunities afforded by the UNCRPD, and promote access to health, well-being and participation, many persons with disabilities, chronic illness or frailty rely on the use of assistive technologies.^14-17^ Assistive technology (AT) is a generic term given to systems supporting the provision, use and evaluation of assistive products. Assistive products are “any product (including devices, equipment, instruments and software) either especially designed and produced, or generally available; whose primary purpose is to maintain or improve an individual’s functioning and independence, and thereby promote their well-being” (p. 2229).^18^ Common examples of assistive products include wheelchairs, prosthetic and orthotic devices, white canes, software for magnification, hearing aids, speech synthesizers and communication boards; robotics, exoskeletons and a range of smart devices are also considered assistive products. Approximately one billion people in the world use assistive products, with the number expected to increase to 2 billion by 2050.^19^ Assistive products can be applied across all domains of an individual’s life, and may promote or enable daily self-care, access to education and employment, participation in civil society and social engagement. They are therefore crucial to support individuals’ quality of life, their rights enshrined in the UNCRPD, and act to break the cycle of exclusion from society. Unfortunately, access to assistive technologies is not uniformly distributed on a global scale, and many factors remain which prevent individuals from accessing these products, services, and systems. This is particularly true in less-resourced settings, where there may be financial or systemic barriers to accessing affordable, accessible, and appropriate assistive products and systems.^20,21^

 Given the critical role of AT it is important to understand how their use and provision has been impacted by the COVID-19 pandemic and to what extent such technologies may also help to mitigate the effects of the pandemic. Understanding the impacts across a variety of settings and cultures may help to identify ways to build resilient services and systems capable of operating within the context of a global health crisis. Therefore, the objectives of this study were to explore the how AT use and provision have been affected during the initial stages of the COVID-19 pandemic, and how AT policies and systems may be made more resilient based on lessons learned during this global crisis.

## Methods

###  Research Design

 This study was a rapid international online qualitative survey in the 6 languages of the United Nations (UN): English, French, Spanish, Russian, Arabic, and Mandarin Chinese. Translations were completed from English by native speakers in each of the 5 additional languages and then back translated to ensure accuracy and consistency. Qualtrics software was used for Internet-mediated distribution in all 6 languages. The survey was conducted over a period of 4 weeks. Reminders were sent twice during this period.

###  Participant Recruitment

 Participants were recruited who were AT users, service providers, or individuals otherwise involved in the use or provision of AT (ie, distributors, researchers, educators). Participants were recruited primarily through the Global Cooperation on Assistive Technology (GATE) Listserv, a global network of AT users, professionals, and researchers managed by the World Health Organization’s GATE initiative, and through the International Disability Alliance network. Investigators and respondents also shared the invitation to the survey within their local and regional networks. Invitations included information regarding the objective of the research, researchers and their affiliations, and ethical approval for the study. Participants were provided with a link to access the survey.

###  Survey Description

 The survey was conducted using a university-licensed version of Qualtrics software, capable of presenting questions in all 6 UN languages. The survey consisted of demographic questions including participants’ role (one or more of AT user, provider, other), country of residence or work, age range, and inclusion in a COVID-19 related vulnerable group due to age over 60, presence of chronic conditions, or weakened immune systems. Participants were also asked to self-identify if they were considered vulnerable for other reasons, and to provide any relevant details Following demographic questions, participants were asked the following open-ended questions (responses from the questions indicated with an asterisk are reported elsewhere). No limits were placed on response length.

1. In the context of COVID-19, what do you think are the key challenges for delivery of AT services?
2. In the context of COVID-19, what do you think are the key challenges regarding the use of assistive technologies, or AT services?
3. What specific suggestions do you have regarding how AT services can be made more resilient in the current situation?
4. Please describe how your daily life circumstances have changed because of COVID-19.
5. What role can digital and assistive technologies play in helping people continue to meaningfully participate in their communities during the current situation?

###  Analysis

**Figure 1:**
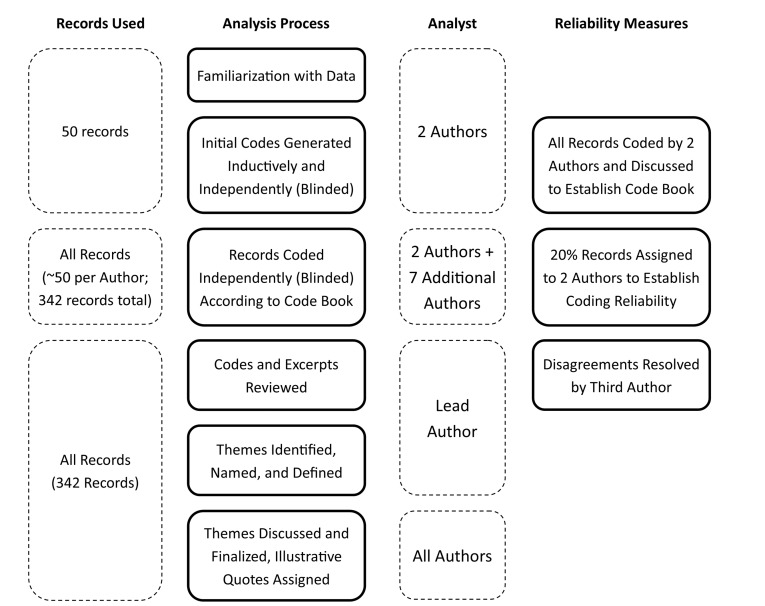
Schematic of Thematic Analysis and Reliability Measures.

 Responses were included in analysis if they had completed a minimum of one of the 5 qualitative questions. Prior to analysis, non-English responses were translated into English by a native speaker of each of the respective survey languages. Demographic data were analyzed using descriptive statistics (ie, counts and percentages). The remaining data were analyzed as qualitative data, using Braun and Clarke’s 6 phased thematic analysis: familiarization with data, generating initial codes, searching for themes, reviewing themes, defining and naming themes, and producing a report.^22^ Following familiarization with the data, initial codes were generated inductively from 50 responses by 2 researchers (EMS and IDE) and reviewed collaboratively to determine a standard set of codes (code book) for each question.^22^ The remaining records were coded by the original 2 coders (EMS and IDE) and 7 additional authors (KLB, MLT, JD, GB, BO, ES, NI) according to the code book, using an online qualitative analysis software (Dedoose). Approximately 20% of records were coded by 2 authors to establish coding agreement as a measure of reliability. Authors were in agreement on codes assigned to approximately 48% of coded excerpts. Of those excerpts which were not in agreement, approximately 75% were excerpts coded differently by 2 authors; the remaining 25% were excerpts coded by one author but not by another. Codes which differed between authors were reviewed by a third author to determine if codes applied to the excerpt. This review resulted in combining of some codes where there was overlap in content. Following completion of coding, codes were reviewed and aggregated into distinct themes and subthemes, defined, and assigned illustrative quotes which are used to present the results. Themes were selected to ensure the majority of data were represented by the themes and subthemes, without substantial overlap between thematic content. Figure 1 outlines the process of thematic analysis and reliability measures.

 Strategies to maintain trustworthiness were used to enhance credibility, dependability, transferability, and confirmability of the data. In particular, a range of coders with differing expertise were engaged to complete analysis to address potential for bias. As described, there was also a process to establish coding reliability. Quotes are presented with themes to enhance credibility of the analysis.

## Results

###  Participant Demographics

**Figure 2:**
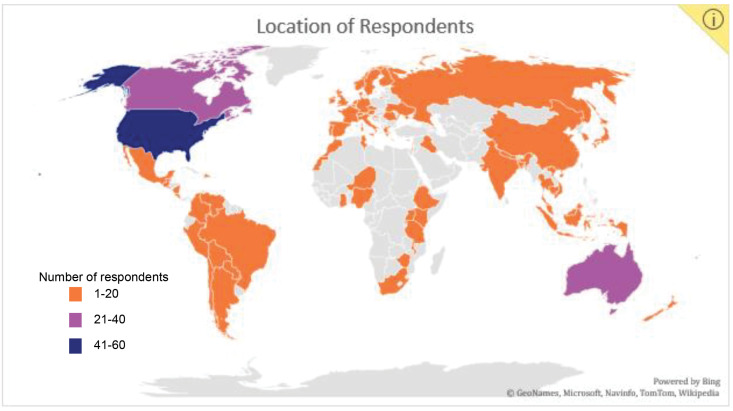
Location of Respondents.

 A total of 342 participants from 72 countries completed at least one question on the survey; 275 participants completed all survey questions. Location responses were categorized by country and 2019 Human Development Index category to provide context for the range of responses.^23^ Demographic and language data are presented in Table. Figure 2 provides a visual representation of the number of responses per country.

**Table:** Respondent Demographics

**Demographic Factor**	**n (% Respondents)**
Role^a^	
AT user	106 (31.0%)
AT provider^b^	209 (61.1%)
Other role related to AT^c^	93 (27.2%)
Location Human Development Index^d^	
Very high	224 (65.5%)
High	55 (16.1%)
Medium	47 (13.7%)
Low	13 (3.8%)
Age^e^	
19-29	23 (6.7%)
30-39	79 (23.1%)
40-49	89 (26.0%)
50-59	83 (24.3%)
60-69	57 (16.7%)
70-79	8 (2.3%)
Member of COVID-19 Vulnerable Group^f^ (Percentage of respondents per role)	
AT user	57 (53.8%)
AT provider	52 (31.1%)
Other role related to AT	53 (39.3%)
Survey language	
English	270 (78.9%)
Spanish	55 (16.1%)
French	10 (2.9%)
Russian	3 (1.0%)
Chinese	1 (0.3%)
Arabic	3 (1.0%)

Abbreviations: COVID-19, coronavirus disease 2019; AT, assistive technology. ^a^ Total exceeds 100% as 61 respondents selected more than one answer. ^b^AT Provider included clinicians, teachers, assistive technology specialists and providers. ^c^Other roles included administrator, academic, educator, AT developers and manufacturers, other healthcare professionals, policy-makers, and researchers. ^d^Three respondents did not provide location and could therefore not be categorized. ^e^Three respondents did not provide age. ^f^Two respondents did not indicate whether they belonged to a member of a vulnerable group.

###  Thematic Analysis

**Figure 3:**
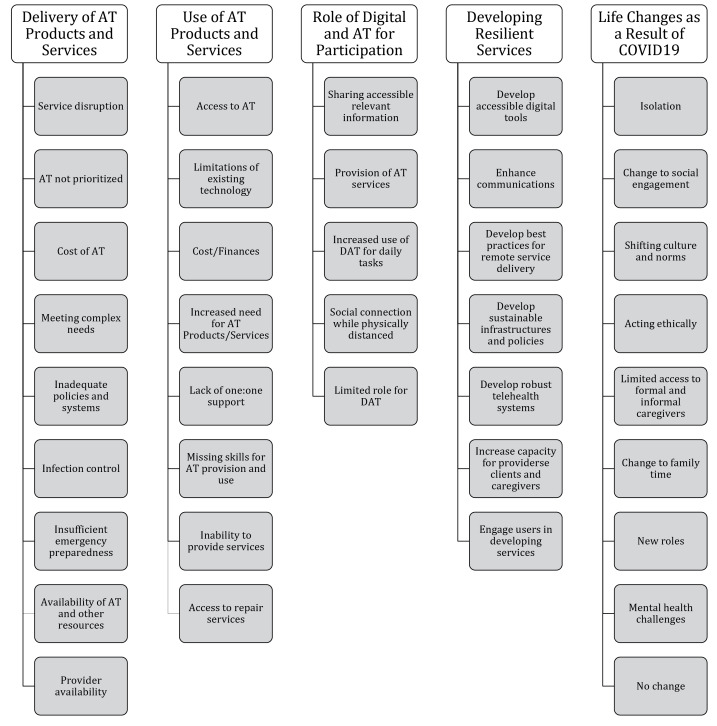
Initial Coding Tree. Abbreviations: COVID-19, coronavirus disease 2019; DAT, digital and assistive technology; AT, assistive technology.

 Four themes were identified in the responses which outline the challenges posed to AT users and providers during the COVID-19 pandemic: Disruption of Services, Insufficient Emergency Preparedness, Limitations in Existing Technology, and Inadequate Policies and Systems. Figure 3 shows a map of initial codes (Code Book) established and defined through inductive coding and applied in deductive coding.

 In the sections below, each of the themes and subthemes (identified in italics) will be described using illustrative quotes. Respondents who were quoted are identified by their role (ATU: AT user, SP: service provider, O: Other), and by their region, classified according to the World Health Organization (WHO) Health regions (AM: Americas, AF: Africa, EU: Europe, SEA: South-East Asia, WP: Western Pacific). Within each theme, one or more subthemes are identified which represent learnings from the experience, and opportunities to move to more resilient services in the future.

####  Disruption of Services

 The theme *Disruption of Services *describes a disruption to the AT services which were being provided or accessed prior to the COVID-19 pandemic. This includes assessment, delivery, training, support, maintenance, and repair services. This disruption was described on some level by all respondents to the survey, regardless of role or geographical location. One respondent described the wide ranging difficulties they were experiencing as a researcher in AT from the Eastern-Mediterranean region, listing “restrictions in face-to-face sessions for supporting users, no access to AT services, assessment centres, and/or AT education services by users…, no easy access to services delivered in hospitals, [and] cancellation of rehab sessions.”

 Despite disruption of services having several root causes, the challenge mentioned most frequently was the *Lack of Provider Availability *and consequent *Lack of One-to-One Support.* Challenges with provider availability were associated with different factors such as providers’ illness, implementation of lockdown measures, and redeployment of providers to other areas of health services. In some cases, the reduction in services resulted in “providers [being] overwhelmed by requests, aggravated by staff reduction during the quarantine period (ATU, EU).” As a consequence, providers reported struggles in providing appropriate services, “while maintaining adequate social distance. Since we need to be able to carry out the service, know the user, be able to carry out the evaluation, take measurements, test, and adjust … without being able to have contact with the person (SP, AM).”

 AT users noted the specific *Lack of One-to-One Support* as a result of the pandemic. This inability to access dedicated support for individuals had impacts on AT users’ ability to use their technology, including acquiring critical skills for independence. This was described as particularly challenging, as “even with the AT some deafblind people still rely on a communication partner… to help them use the devices to communicate (O, SEA).” Furthermore, “for those who require a support worker to assist with use of AT, there may be an issue of getting suitable support and training support workers to assist the user (SP, WP).”

 The focus on *Infection Control *while delivering services may be one underlying cause of difficulties accessing providers and one-to-one support. As one AT user noted, “the fact that COVID-19 can remain on anything including the assistive device makes easy potential victims to acquire the virus (ATU, AF).” As a result of infection control measures, including isolation, service providers described “not being able to interact with at-risk patients due to risk of cross-contamination, [or to] access patients to provide appropriate access to technology (SP, AM).” For many providers this meant “balancing risk of physically attending services and clinics with risks of virus exposure (SP, EU).” Unfortunately, in many cases these infection control measures have not been translated into evidence-based guidelines for service provision. As one respondent noted, “this deficiency forces extreme measures in an abundance of caution, therefore service providers must restrict access to ensure that most users are not put at an additional risk (SP, AM).” However, while this may “superficially [be] to protect the vendor and the recipient from infection, [it] has the consequence of a human rights infringement (O, WP).”

 This lack of provider availability and one to one support may have been exacerbated by *AT Not Being Prioritized *as an essential service. One respondent highlighted the need for “consideration of the essential nature of assistive technologies for the survival of persons with disabilities and the maintenance of their quality of social participation, even in times of confinement (ATU, AM).” The designation of AT as an essential service may have resulted in fewer redeployments and an increased availability of service providers.

 To address these challenges, and develop resilience for future global emergencies, respondents noted the need to *Develop Best Practices for Remote Service Delivery.* These would include development and evaluation of new service delivery models, and access to the necessary equipment including personal protective equipment, and include a need to “develop systems that support remote assessment, prescription, fitting, and follow-up, [as well as] reporting and data collection systems (O, AM).” This was further described as a need “to do things differently… We need to tap into technology to do evaluations/fittings, and use personal protective equipment to go into homes for things that cannot be done via technology (SP, AM).” The effort to develop novel best practices for service delivery in similar circumstances would ensure “preventative measures are known and applied [resulting in] developing the capacity of the sector to a ‘digitally transformed’ service mode (SP, EU).” Best practices should equally address remote service delivery, as well as in-person services where they are necessary or unavoidable.

 In developing these best practices, respondents highlighted that governments and other responsible institutions should focus on the importance of *Engaging Users.* Participatory and iterative engagement with AT users is viewed as essential in understanding their needs and develop person-centred services. This could take the form of “direct involvement of leaders from user groups (O, SEA),” and may include “co-creation processes for the suggestion of alternatives for AT services in cases of similar settings (SP, EU).” Regardless of the method, respondents were clear that future planning must “involve the beneficiaries themselves to understand the gaps and their needs (SP, AF).” This focus on user-centred development should apply equally to service models, as well as novel technologies, policies and systems.

####  Insufficient Emergency Preparedness

 The disruption of services experienced by both AT users and providers may be due, in part, to* Insufficient Emergency Preparedness* within the AT ecosystem leading up to the pandemic. One respondent, an AT user working for a large national health service, reported an “inability to link up several different organizations to allow delivery of AT, during time of great upheaval” and further described “putting in place emergency planning for our own organization but not yet liaising with other linked organizations, [or] identifying new protocols (ATU/SP, EU).” In circumstances where governments moved quickly to enact lockdown measures, some clients were ill prepared to access resources, or could not access the necessary technologies to participate in virtual or telehealth-based service delivery. One participant pointed out how “technical support will be the primary issue. While it may be that some people have access to friends who can help, this may not always be the case (ATU, WP).”

 In many cases, clients, their caregivers, and even service providers were *Missing Critical Skills* to use their assistive technologies or support technologies. One respondent envisioned there would not be “training and awareness for sometime. Some may access services online through emails, phone calls, but the rural population will miss out and suffer a lot. To a certain extent, clients may even stop using their assistive products (SP, WP).” For providers, remote service provision may be possible, but it requires both themselves and their clients “to have some level of tech experience (SP, AM).” Unfortunately, as another respondent pointed out, “many of the people we serve are not as ready to use virtual means of communication (O, AM).”

 To address these challenges, respondents suggested it would be necessary to *Increase Capacity for Providers, Clients, and Caregivers.* This could take the form of providing training in use, maintenance, and troubleshooting of the AT directly to users and their caregivers, who could then, if necessary, in turn “provide … support to the AT users. This can extend beyond parents to include siblings, cousins etc that are in the home with the AT users. Additionally, professionals can increase their virtual outreach to users and families to identify specific needs so that support can be individualized and thus more effective (O, AM).” This process could be supported through the establishment of communication channels to “ensure support and technical issues can be addressed on a regular basis and heightened anxieties reduced (SP, EU).” Addressing the necessary skills for clients to receive remote service provision is critical to ensuring a robust system.

####  Limitations in Existing Technology

 Despite best efforts to address some of the challenges associated with provider availability and limited support, respondents identified many *Limitations in Existing Technology *which continued to challenge their access to use or provide services. This theme identifies the need to have technologies which are capable of supporting best practices for remote service delivery during pandemic situations, as well as communicating critical health and social information with citizens in effective and accessible ways. There are a variety of existing technologies to support remote communication which can be used for the delivery of AT services, however the use of these “is much more difficult for less technologically advanced people and those without laptop computers or smart phones, as well as those who cannot afford these technologies (SP, EM).” In particular, one respondent felt “people who are visually, auditory, or intellectually impaired have a harder time with the technology (SP, EM),” and noted that sometimes there are additional cultural barriers for their use. The consequence of an inability to access or use these technologies is “uncertainty on what we understand (ATU/SP, EU),” in terms of public health guidelines, mechanisms for accessing services and appropriate measures to stay safe during a pandemic situation.

 In particular, there is a challenge *Meeting Complex Needs *of clients who may have alternative methods of accessing information and services. Take, for example, the experience of someone who is deafblind. As one respondent indicated, “access to [COVID-19 related] information is a key challenge due to a lack of information in accessible format, … Social distancing is going to reduce already limited tactile sign language support, critical for them (O, SEA).” For many people with more complex needs, the pandemic situation has resulted in them “having no option but to make do, with AT that doesn’t suit [our] needs (SP, WP).”

 In order to address this challenge, respondents suggested a need to *Develop Robust and Accessible Tools and Systems*, which would be capable of delivering information and AT related services at a distance. This could take the form of “increased use of digital platforms to allow for improved ability to assess and make appropriate recommendations (SP, AM).” Respondents suggested it would be important to encourage “consistency between AT and digital technologies to ensure interfacing and universal access of AT. Combining what is available in the mainstream world and what is available within the AT world will create resilience because our technology can be adapted (SP, SEA).” This focus on developing universally accessible systems which are compatible with AT used by people with disabilities was echoed by another respondent who advocated for inclusively designed digital technologies, and provided an example of “visually impaired people being put on special leave, even though they are willing and able to work, due to poorly-designed remote services (ATU, WP).” This may exacerbate issues of exclusion from society and further marginalize a population already at risk.

 However, the development and use of these tools requires a considered approach. In the words of one respondent, increased reliance on digital technologies would require “rapid upskilling in telehealth triaging (O, WP).” Furthermore, another participant highlighted how it is important to consider “what is actually feasible via ‘telerehabilitation’ (O, AM).” In addition to further development of the technology and skills to support it, there is a need for “legislative support, integration into health registration bodies, and training (SP, WP).” It is also important to consider “alternative ways to deliver information, not all online given that some people don’t have access to or knowledge of how to access online… services (SP, AM).” While developing these strategies and technologies, it is crucial to put “the rights of people with disabilities at the forefront (SP, AM),” as it would be a key factor in the successful adoption of these newly developed systems.

####  Inadequate Policies and Systems

 While the previous themes identified a need for robust telehealth systems and digital tools, and best practices to support the delivery of remote services, this theme focuses on the policies and systems which must be in place for these tools to be used effectively. The theme *Inadequate Policies and Systems* discusses the extent to which delivery of and access to services is challenged by inefficient, inadequate, or missing policies and systems. As a result of the pandemic, respondents to our survey reported an *Increased Need for AT Products and Services* to help individuals manage their situation at home during periods of isolation. Specifically, there was a noted need to increase “deployment of support technologies to facilitate the autonomy of people who have a disability (ATU, AM)”. However, at the same time, many respondents reported *Difficulty Accessing AT, *due to shortages, challenges with transportation, or lack of service providers. This has resulted in people being “able to use existing AT, but not able to obtain new AT (SP, AM).” For many children who typically receive services in school, “the closure of schools is preventing the use of AT for communication and learning for children whose families cannot provide these at home due to poverty (SP, AF).”

 Notably, the *Cost of AT* has presented a problem during these times, particularly where level of income might have decreased further as a consequence of the pandemic situation. As one respondent described, “loss of employment for many means less income to afford AT, repairs, or training/support to use it. This could result in further digital isolation (ATU, WP).” In fact, some respondents looked towards the future, noting “the economic contraction which has already started will be an enormous obstacle to any AT which is not entirely free (ATU/SP, AM).”

 Cost is not only limited to funding of the technology itself. In many cases, respondents discussed issues of funding by health systems for remote service delivery. According to one respondent, “even with the right technology, insurance reimbursement is not set up … to provide telehealth services (SP, AM).” This may be even more challenging in lower to middle income countries, where “travel bans are preventing the donation, provision, and servicing of AT by foreign non-governmental organizations (SP, AF),” who often act as the primary service provider. Furthermore, delivery of remote or telehealth services relies heavily on access to the Internet through available technology. As one provider noted, “if someone has the Internet, equipment and software and access method already, AND is proficient one can provide support and training remotely. However, this is not usually the case (SP, AM).” Lack of access to appropriate digital technologies and infrastructure, including consistent access to the Internet, were mentioned regularly as barriers to remote AT services by respondents in all regions.

 Ensuring equitable access to AT products and services requires the *Development of Sustainable Funding Infrastructures and Policies. *Respondents provided a variety of suggestions as to how this might be accomplished, including providers “being recognized as an essential service by the public health authority, in order to assure full functionality of the AT system in case of a shutdown (SP, EU).” Achieving continuity of AT provision, and ensuring the delivery of services through remote means would require “flexibility in funding body policies allowing alternative service provision (SP, WP).” However, simply addressing access to service providers may not be sufficient. It was suggested it is necessary to “recognize the needed wraparound AT services to support the product, recognizing the collateral processes [including] freight, borders, regulations, and signoffs which are removable barriers in times like these (O, WP).” Each of these considerations feeds into the need for sustainable funding infrastructures and policies.

## Discussion

 As with other sectors of society, the COVID-19 pandemic has had wide reaching impacts on both the use and provision of assistive technologies. These include both access to the products and to related services. COVID-19 has exposed challenges with existing AT systems which must be addressed to build resiliency. Our study has identified a substantial disruption of services, largely associated with availability of service providers and one-to-one support due to infection control measures. We were also able to identify a range of approaches to build resilient systems for AT at the national and global level, through the development and evaluation of sustainable policies and systems, and robust accessible technologies.

 In line with international commitments to the UNCRPD and recent WHO resolutions, previous research and our current work, it is clear AT must be included as part of universal health coverage, and understood to be an essential service.^24,25^ This will require appropriate protocols to be put in place to deliver services safely and effectively, according to a rights-based approach, during times of crisis. While COVID-19 was the catalyst to reveal deficiencies within our preparedness, technologies, policies, and systems, addressing each of these issues more systemically and in non-pandemic times would be to the benefit of all AT users.

 There have been incredible and rapid changes made throughout the COVID-19 experience in terms of remote service delivery, required regulations and repurposing of existing technology to meet service delivery needs.^26^ However, it is clear these technologies, services, and approaches have been unable to fully meet the needs of all, and have further widened issues of inequality with more vulnerable persons being often left behind. Progressively moving services from physical to digital will require coordination, development of infrastructural resources and upskilling for both service providers and users.^27^ It will necessitate better working across disciplines and taking a more challenge-based approach to break down siloed activity. COVID-19 has provided an opportunity to rapidly assess the application of digital service delivery. We must now learn from the experience, while taking care not to regress to the point where it was seen as impossible to deliver these services at a distance. We must also go a step further, ensuring that those who are most impacted by changes to service delivery – AT users – are at the centre of the conversation, and engaged in the development of user-centred technologies, systems, and policies.^28,29^ In some cases digital technologies can replace aspects of physical service provision, but in many cases they will augment rather than replace. However, such augmentation or replacement is only desirable if it is not susceptible to greater disruption in a crisis situation.

 It is important to also consider the broader context of digital service delivery and ensure this does not further marginalize populations who may not have consistent access to the Internet, or the technologies required to access it. Mobile access and use is key to this,^30^ as mobile is often the first interaction people have in lower income contexts have with the Internet, but disabled people struggle to harness Internet due to barriers including cost and accessibility.^31^ Among those issues highlighted or exacerbated by the COVID-19 pandemic is the digital divide, between those who have access to digital technologies and reliable Internet, and those who have not.^32,33^ While this digital divide has been documented in lower income contexts,^30,34^ it is also apparent in high income contexts, where individuals of lower socio-economic status, or those in rural and remote areas, may still struggle with access to technological infrastructure and reliable Internet. During this crisis, as more and more individuals have been asked to stay home, for work, education, and social activities, access to the Internet and the technology required to access it have become even more important.^35^ In some contexts, this has fueled further discussion about access to the Internet, and consequently access to information, education, work, and social engagement during pandemic times, as a human right.^36,37^

 While there was general agreement in the themes regarding the challenges faced by AT users and providers in the context of COVID-19, there were no examples presented of individuals who were experiencing no change to their service use or provision, nor examples of excellent policies or systems. As this study was conducted early in the pandemic, this may result from initial systematic challenges in meeting the needs of populations in a crisis. This may also be due to a self-selection bias where individuals who were experiencing difficulty may have been more likely to respond to the survey.

 There were insufficient responses to provide subgroup analysis by region or income level, with only one fifth of respondents being outside countries with very high or high Human Development Index scores, or to evaluate differences by type of health system or access to AT in non-pandemic times. However, challenges which were identified were similar regardless of the country of the respondent. Challenges with inadequate policies and systems, and a lack of emergency preparedness have had similar effects in terms of lack of provider availability, and one-to-one support. The lack of adequate policies and systems for AT provision have been documented globally, therefore this is not surprising.^29,38^ It is important to note pre-existing inequitable access to these products and services prior to the pandemic, across contexts, and regardless of health system type. This highlights the challenge of intersectionality across disability and socioeconomic status.^39^ Individuals who were disadvantaged prior to the pandemic likely experienced further disadvantage as a result of COVID-19.

 The study was deployed during the peak of the first wave of the pandemic in Europe, after significant progress had been made in other areas (eg, China) and before significant increases in cases in the Americas, Africa, and South East Asia. Results are therefore reflective of the time in which the survey was deployed. There is an opportunity for future research to assess the long-term impacts of COVID-19 on AT provision. Where changes have been made to service delivery systems as a result of lessons learned in the current pandemic, there may also be an opportunity to assess comparative outcomes, and the inclusion of AT users in policy development and implementation processes.

###  Limitations

 The use of an online survey may have limited responses to those who have access both to the Internet, and to the required technology to access it. This may partially explain the divide in terms of our responses across income and development contexts and may have resulted in a much higher number of responses in English. This was mitigated by distributing the survey in all 6 UN languages, however the networks used for distribution exist primarily in English, and therefore may not include all who would have been interested or eligible to respond. This may have resulted in a lower response rate in lower-resourced environments, therefore the results may not be generalizable across these contexts. The study was also distributed through official channels and may not fully represent the global population of AT users and providers. Furthermore, the study was conducted early in the pandemic and may not be reflective of the longer-term effects of COVID-19 on AT use and provision.

## Conclusion

 COVID-19 has had specific impacts on the delivery of AT services, primarily due to infection control measures resulting in lack of provider availability and diminished one-to-one services. To build resilient systems capable of addressing AT users’ needs, both during and outside of pandemic times, the study concludes with some key recommendations in Box 1. Each of these should be developed within a rights-based approach and universal health coverage system which recognizes AT as an essential need.

**Box 1.Specific Actions to Promote AT Resilience in Crisis Situations**

1. Service users must be engaged in development of sustainable policies and infrastructures which meet their needs using a co-created rights-based approach.
2. Service providers must develop their skills and capacity for delivery of remote services and provide training and appropriate digital and assistive technologies to clients and their caregivers to facilitate engagement with health services and their communities.
3. Product manufacturers must work with service users to understand their needs for digital and assistive technologies, and develop robust and accessible tools for service delivery.
4. AT must be recognized as an essential service, and must be accompanied by best practices and funding models for remote service delivery.
5. Policies and infrastructures must be developed which recognize access to AT as key to the realization of rights for persons who require them and must include AT within a universal health coverage system.

Abbreviation: AT, assistive technology.

## Acknowledgements

 The authors would like to acknowledge Chao Bian for his assistance translating the survey into Mandarin Chinese, and for translating responses back into English.

## Ethical issues

 Each participant was provided information regarding their participation including information about the study objectives, researchers, and ethical approval, as well as any potential risks and benefits, prior to consenting to participation. Consent was assumed to be given for participation if the participant proceeded to the first page of questions. Ethical approval for the survey was granted by Maynooth University’s Social Research Ethics Committee (#2402124).

## Competing interests

 Authors declare that they have no competing interests.

## Authors’ contributions

 EMS: Conception and design, acquisition of data, analysis and interpretation of data, drafting of the manuscript, statistical analysis. MLTH: Conception and design, acquisition of the data, analysis and interpretation of the data, drafting of the manuscript. IDE: Conception and design, analysis and interpretation of the data, drafting of the manuscript. EVS, GB, KLB, JD, BO, and NI: Analysis and interpretation of the data, drafting of the manuscript. CH: Critical review of the manuscript for important intellectual content, supervision. MM: Conception and design, critical review of the manuscript for important intellectual content, supervision.

## Funding

 EMS, GB, JD, BO, and CH are supported by Assistive Technology 2030, funded by UK Aid and delivered by GDI Hub. EMS is supported by a Canadian Institutes of Health Research Fellowship. IDE is supported by the Irish Research Council. KLB is supported by the Fonds de Resecherce Sante de Quebec.

## Authors’ affiliations

^1 ^Assisting Living and Learning Institute, Maynooth University, Maynooth, Ireland. ^2^Independent Researcher, Madrid, Spain. ^3^Athena Institute, Vrije Universiteit, Amsterdam, The Netherlands. ^4^Global Disability Innovation Hub, University College London, London, UK. ^5^Center for Interdisciplinary Research in Rehabilitation and Social Integration, Universite Laval, Québec, QC, Canada. ^6^Centre for Global Health, Trinity College Dublin, Dublin 2, Ireland. ^7^University College London Interaction Centre, University College London, London, UK. ^8^Assisting Living and Learning Institute, Department of Psychology, Maynooth University, Maynooth, Ireland.
